# Danish Integrated Antimicrobial Resistance Monitoring and Research Program

**DOI:** 10.3201/eid1311.070421

**Published:** 2007-11

**Authors:** Anette M. Hammerum, Ole E. Heuer, Hanne-Dorthe Emborg, Line Bagger-Skjøt, Vibeke F. Jensen, Anne-Marie Rogues, Robert L. Skov, Yvonne Agersø, Christian T. Brandt, Anne Mette Seyfarth, Arno Muller, Karin Hovgaard, Justin Ajufo, Flemming Bager, Frank M. Aarestrup, Niels Frimodt-Møller, Henrik C. Wegener, Dominique L. Monnet

**Affiliations:** * Statens Serum Institut, Copenhagen, Denmark; † Technical University of Denmark, Copenhagen, Denmark; ‡ Danish Medicine Agency, Copenhagen, Denmark; § Danish Veterinary and Food Administration, Søborg, Denmark

**Keywords:** Antibiotics, resistance, humans, epidemiology, DANMAP, perspective

## Abstract

Resistance to antimicrobial agents is an emerging problem worldwide. Awareness of the undesirable consequences of its widespread occurrence has led to the initiation of antimicrobial agent resistance monitoring programs in several countries. In 1995, Denmark was the first country to establish a systematic and continuous monitoring program of antimicrobial drug consumption and antimicrobial agent resistance in animals, food, and humans, the Danish Integrated Antimicrobial Resistance Monitoring and Research Program (DANMAP). Monitoring of antimicrobial drug resistance and a range of research activities related to DANMAP have contributed to restrictions or bans of use of several antimicrobial agents in food animals in Denmark and other European Union countries.

## Abstract 2

This program has led to changes in the use of antimicrobial agents in Denmark and other countries.

In 1969, the Swann Committee recommended to the British government that antimicrobial agents used for human therapy, or antimicrobial substances that selected for resistance to these agents, should not be used for growth promotion in food animals (*1*). In 1993, the first report of nonhospital and nonhuman reservoirs of *vanA* vancomycin-resistant *Enterococcus faecium* (VREF) in the United Kingdom was published (*2*). This finding was surprising because no therapeutic glycopeptide (vancomycin or teicoplanin) had been used in food animals. However, another glycopeptide, avoparcin, had been used for decades as a feed additive for growth promotion; it was suggested that the occurrence of VREF might be related to this usage. The finding of VREF in the United Kingdom led to similar investigations and subsequent findings in Germany and Denmark in 1994 and 1995 (*3*,*4*). In 1995, one of the only antimicrobial agents available for treatment of multidrug-resistant enterococci and methicillin-resistant *Staphylococcus aureus* (MRSA) infections was vancomycin. On May 18, 1995, the Danish Minister of Agriculture and Fisheries banned the use of avoparcin nationally because new scientific evidence showed that avoparcin used as a growth promoter in food animals constituted a potential threat to human health (Article 11 of Council Directive 84/587/EEC). In July 1995, the ban became effective in all countries in the European Union (EU) after a decision by the EU Council of Ministers.

The avoparcin ban called attention to the wide array of antimicrobial substances being used in food animals for growth promotion or disease control and to the risk for transfer of other resistant bacteria or resistance genes from animals to humans through the food chain. A systematic approach was needed to generate the data necessary to determine the magnitude of current or potential future public health hazards from nonhuman use of antimicrobial agents. In September 1995, the Danish Integrated Antimicrobial Resistance Monitoring and Research Program (DANMAP) was established at the initiative of the Danish Ministry of Health and the Danish Ministry of Food, Agriculture and Fisheries, as a coordinated national monitoring and research program. Participants in the program are Statens Serum Institut, the National Food Institute and National Veterinary Institute at the Technical University of Denmark, the Danish Veterinary and Food Administration, and the Danish Medicines Agency. DANMAP has 4 objectives: 1) monitor the consumption of antimicrobial agents for food animals and humans; 2) monitor the occurrence of antimicrobial agent resistance in bacteria isolated from food animals, food of animal origin, and humans; 3) study associations between antimicrobial agent consumption and antimicrobial agent resistance; and 4) identify routes of transmission and areas for further research.

Denmark was the first country to establish systematic and continuous monitoring of antimicrobial agent consumption and resistance in animals, food, and humans. Other antimicrobial agent resistance monitoring programs are now established in other countries: Norway (Usage of Antimicrobial Agents and Occurrence of Antimicrobial Resistance in Norway [NORM/NORM-VET]) (*5*), Sweden (Swedish Veterinary Antimicrobial Resistance Monitoring [SVARM] and Report on Swedish Antibiotic Utilisation and Resistance in Human Medicine [SWEDRES])(*6*), the Netherlands (Monitoring of Antimicrobial Resistance and Antibiotic Usage in Animals in the Netherlands [MARAN] and Consumption of Antimicrobial Agents and Antimicrobial Resistance among Medically Important Bacteria in the Netherlands [NETHMAP]) (*7*,*8*), Canada (Canadian Integrated Program for Antimicrobial Resistance Surveillance [CIPARS]) (*9*), and the United States (National Antimicrobial Resistance Monitoring System. [NARMS]) (*10*).

The first results covering all 3 reservoirs from DANMAP were published in 1997 (*11*); annual reports have subsequently been published and are available at www.danmap.org. We present selected results and experiences from 11 years of monitoring and reporting of antimicrobial agent consumption and antimicrobial agent resistance in bacteria isolated from animals, food, and humans in Denmark.

## Description

**Figure 1:**
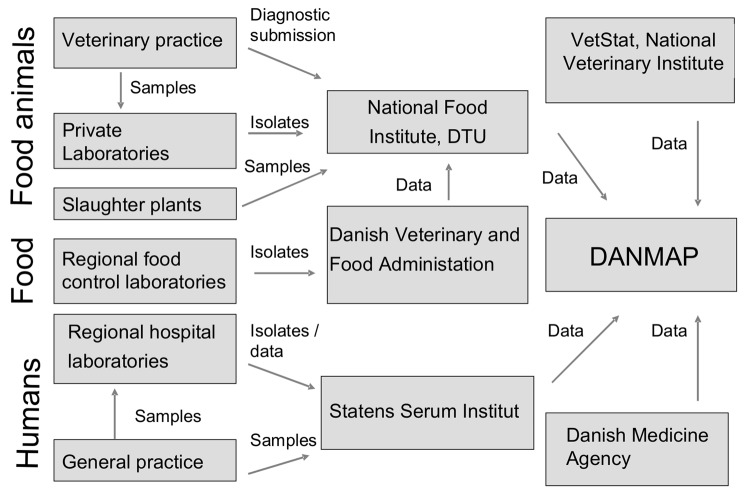
Data flow. DANMAP, Danish Integrated Antimicrobial Resistance Monitoring and Research Program; DTU, Technical University of Denmark.

DANMAP collects and presents data on consumption of antimicrobial agents and the occurrence of resistance in indicator bacteria, zoonotic bacteria, and pathogenic bacteria from animals, food, and humans. The setup for sampling of isolates and data for DANMAP are briefly described below. A schematic description of sampling of isolates and data flow is presented in Figure 1. A more detailed description can be found in the DANMAP reports (www.danmap.org).

### Isolates from Animals

Bacterial isolates are collected from healthy animals at slaughter (*Escherichia coli*, enterococci, and *Campylobacter* spp.) as well as from diagnostic submissions (*Staphylococcus hyicus* from pigs and *E. coli* from cattle and pigs with diarrhea). *Salmonella* isolates from subclinical as well as clinical cases of salmonellosis are included. *Salmonella* isolates from subclinically infected pigs and poultry are collected on farms as part of a national *Salmonella* monitoring program.

### Isolates from Food

All food samples are collected at wholesale and retail outlets during routine inspections by the Regional Veterinary and Food Control Authorities (*Salmonella* and *Campylobacter* spp.) or on request from DANMAP (enterococci and *E. coli*). Bacterial isolates included in the monitoring program originate from food from Denmark as well as imported food.

### Isolates from Healthy Persons in the Community

To monitor the level of resistance among healthy persons, ongoing surveillance was initiated in 2002. Currently, 1 isolate each of *E. faecium*, *E. faecalis,* and *E. coli* is sampled from each fecal sample, if isolated. Furthermore, a selective method is used to detect vancomycin-resistant enterococci.

### Isolates from Patients in the Community and in Hospitals

Isolates of *Salmonella* and *Campylobacter* included in the monitoring program originate from diagnostic submissions sent to Statens Serum Institut (SSI). For *S. aureus* testing, all blood isolates from 15 of 16 Danish counties and all MRSA isolates nationwide are sent to SSI. For *Streptococcus pneumoniae* testing, all isolates from blood and spinal fluid found nationwide by clinical microbiology laboratories are sent to SSI. For *E. coli,* coagulase-negative staphylococci, and *Streptococcus pyogenes*, data on all isolates from blood samples (*E. coli*, coagulase-negative staphylococci), urine samples (*E. coli*), and clinical samples (*S. pyogenes*) submitted for susceptibility testing are provided by clinical microbiology laboratories participating in the Danish Study Group for Antimicrobial Resistance Surveillance.

## Changes after 11 Years of DANMAP

### Ban of Antimicrobial Growth Promoters

**Figure 2:**
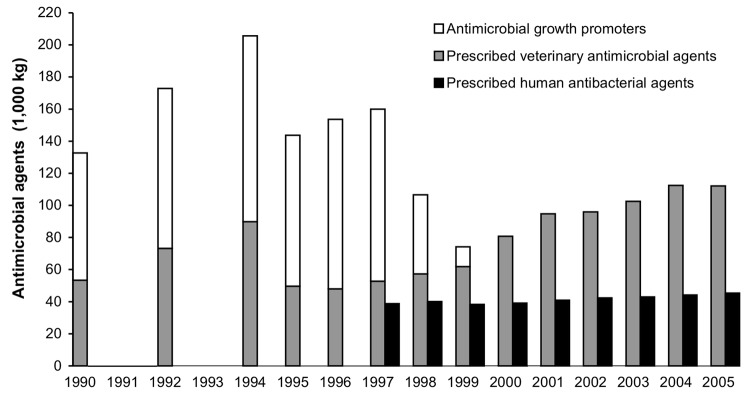
Consumption of prescribed antimicrobial agents and growth promoters in animal production and prescribed antibacterial agents in humans, Denmark, 1990–2005 (*12*).

In 1994, the consumption of antimicrobial growth promoters constituted more than half of the total antimicrobial consumption by animals in Denmark (Figure 2). Antimicrobial growth promoters were used as an in-feed supplement for nearly all broiler chicken and pig farms in Denmark. At the time avoparcin was banned, a high level of resistance to several antimicrobial growth promoters was observed among bacteria from production animals (*11*). Although use of avoparcin was banned in 1995, the total use of growth promoters increased until 1998. In the spring of 1998, the Danish pig and poultry producers voluntarily discontinued use of all antimicrobial growth promoters in finisher pigs and broiler chickens. DANMAP measured and documented the effect of this marked change in antimicrobial consumption in animal production.

**Figure 3:**
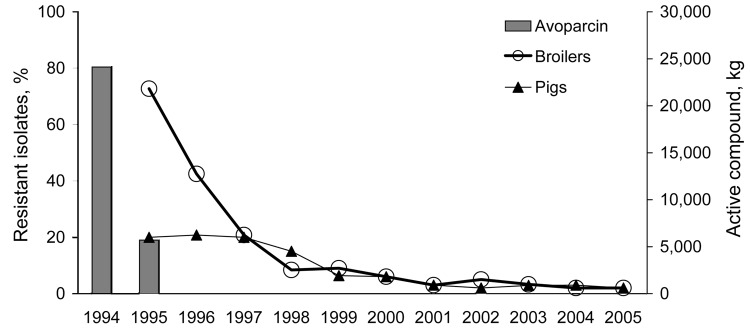
Trends in glycopeptide resistance among *Enterococcus faecium* from broiler chickens and pigs and the consumption of the growth promoter avoparcin in animals, Denmark, 1994–2005 (revised from *12*).

By 2006, 11 years of monitoring data were available (Table; Figure 3) (*12*). The 1995 ban on avoparcin had a substantial effect on lowering the occurrence of VREF isolated from fecal samples from broiler chickens (Figure 3). In 2005, <3% of the *E. faecium* isolates from broiler chickens were resistant to vancomycin (*12*). To avoid bias in the selection of the isolates, the VREF isolates included in DANMAP were detected by using a nonselective isolation method. Although studies that used selective enrichment for isolation of VREF have documented that VREF could still be isolated from a high percentage of poultry flocks several years after the ban of avoparcin (*17**,**18*), the quantity of VREF isolated from Danish poultry has been substantially reduced.

**Table:** DANMAP’s contributions to decreasing antimicrobial agent resistance in Denmark, 11 years*

Sector	Problem	Intervention (reference)	Type of intervention	Intervention had effect
Food animal	High occurrence of vancomycin-resistant *Enterococcus faecium* isolates in food and production animals	Banned avoparcin: Denmark May 1995 and EU Dec 1997. Provided data for national and EU ban. Monitored effect of ban in animals, food, and healthy humans.	Regulatory	Yes
	High occurrence of streptogramin-resistant *E. faecium* isolates in food and production animals	Banned virginiamycin: Denmark Jan 1998 and EU Jul 1999. Provided data for national and EU ban. Monitored effect of ban in animals, food, and healthy humans.	Regulatory	Yes
	High use of fluoroquinolones in animal production	Restricted use of fluoroquinolones in animal husbandry, by Danish law in 2002.	Regulatory	Yes
	High use of antimicrobial agents in swine production	Implemented new guidelines for veterinary practitioner prescription of antibacterial agents for swine production in 2005.	Guideline	Not yet known
Food	Higher levels of resistance in *Salmonella* and *Campylobacter* isolates from imported food than from Danish food	Implemented evaluation of safety of food products, by November 2006.	Regulatory	Not yet known
Human	Increasing macrolide resistance in *Streptococcus pneumoniae*	Published report to prescribers in EPI-NEWS (*13*).	Awareness campaign	Yes
	Increasing use of newer, broad-spectrum antibiotics, especially in hospitals	Published report to prescribers in EPI-NEWS (*14*).	Awareness campaign	No, use of newer, broad-spectrum antibiotics still increasing
	Higher levels of resistance in travel-associated *Salmonella* and *Campylobacter* infections	Published report to prescribers in EPI-NEWS (*14*).	Awareness campaign	Not yet known
	Increasing no. of methicillin-resistant *Staphylococcus aureus* (MRSA) cases	Published report to prescribers in EPI-NEWS (*15*). Informed national reference center, which reported to physicians and other prescribers (*15*). Made notification mandatory Nov 2006 (*16*).	Mandatory notification	Not yet known
	Increasing use of antimicrobial agents, outside and inside hospitals	Published report to prescribers in EPI-NEWS (*14*).	Awareness campaign	No, antibiotic use still increasing
	Increasing ciprofloxacin resistance related to increasing ciprofloxacin use	Published report to prescribers in EPI-NEWS (*14*).	Awareness campaign	No, ciprofloxacin use and resistance still increasing, but low compared with that of other countries

*DANMAP, Danish Integrated Antimicrobial Resistance Monitoring and Research Program; EU, European Union.

In contrast, no significant change in the occurrence of VREF in pigs was observed in the first years after the ban of avoparcin. Nearly all VREF isolated from pigs in Denmark belonged to the same clone; whereas VREF isolates from broilers were polyclonal (*17**,**19*). In the pig VREF clone, genes encoding resistance to glycopeptides (*vanA*) and macrolides (*erm*[B]) were shown to be located on the same mobile DNA element (*19*). The consumption of macrolides (tylosin) for growth promotion decreased substantially in 1998, and a statistically significant decrease in the occurrence of VREF among *E. faecium* isolates from pigs was observed in 1999 and 2000, which suggests that persistence of VREF among the pig population was caused by the continued use of macrolides, mainly tylosin, for growth promotion and therapy.

Since 2002, fecal samples from 485 healthy human volunteers in Denmark have been screened for vancomycin-resistant enterococci. Only 3 VREF isolates (*12**,**20*) and 2 vancomycin-resistant *E. faecalis* isolates have been detected by using a selective isolation method (*21*).

Transfer of VREF from animals to humans can be difficult to demonstrate, especially if only the *vanA* gene encoding vancomycin resistance is transferred. Several animal studies and 1 human study have shown that gene transfer between enterococcal isolates is possible in mouse and human intestines, which indicates that gene transfer can take place in intestines of humans that have eaten meat containing enterococci (*22**,**23*). Transfer of the *vanA* gene is cause for concern if it is transferred to an *E. faecium* isolate belonging to an invasive clone, e.g., clonal complex 17, which causes *E. faecium* infections in humans (*24*).

In January 1998, another antimicrobial growth promoter, virginiamycin, was banned from use in Denmark. Resistance to virginiamycin confers cross-resistance to pristinamycin and quinupristin/dalfopristin, a new antimicrobial with a wide gram-positive spectrum including MRSA and VREF. The Danish ban on virginiamycin was based on the same concerns for human health as the ban on avoparcin. The ban on virginiamycin had an effect also on the occurrence of streptogramin-resistant *E. faecium* in broiler chickens and pigs (*12*).

In July 1999, virginiamycin, together with 3 other growth promoters—tylosin, spiramycin, and bacitracin—was banned in the entire EU. The final step in the termination of the use of antimicrobial agents for growth promotion was taken in December 2002 when the EU Council of Ministers, with a Danish president, decided that all use of antimicrobial growth promoters should be terminated within the EU starting January 1, 2006 (*25*).

### VetStat Monitoring Program

In Denmark, all antimicrobial agents used in animals, except coccidiostats used in poultry, are available by prescription only. To collect information about veterinary use of antimicrobial agents, the Danish Medicines Agency obtained data from the pharmaceutical industry and importers during 1996–2001. These data were used in the first DANMAP reports (1996–2000). In addition, the consumption of antimicrobial agents from 1990 through 1994 was estimated by data collected from the pharmaceutical industry. However, these data did not contain information on antimicrobial agent use within the different animal species. On the basis of recommendations from the EU conference The Microbial Threat, held in Copenhagen in 1998, the Danish government decided that a monitoring system of all veterinary use of prescription medicine on a detailed level should be developed (*26*). The implementation of this monitoring program, VetStat, was initiated in 2000. VetStat data on prescription medicines used in animals are collected from pharmacies, feed mills, and veterinary practitioners. From these 3 sources, detailed data comprising farm identity, species, age group, disease group, identity of medicine, amount, date of purchase, and identity of the prescribing veterinarian are obtained for all antimicrobial agents used in production animals. Valid data by animal species and age group level were reported for the first time in the DANMAP 2001 report.

### Use of Fluoroquinolones in Animal Production

In 2002, the Danish regulation restricted the use of fluoroquinolones, e.g., enrofloxacin, difloxacin, and marbofloxacin, in animal husbandry. Fluoroquinolone use is legal in food animals only when susceptibility tests performed in an approved diagnostic laboratory show that the infecting bacteria are resistant to all other antimicrobial agents registered for treatment in the animal species concerned (*27*). Subsequently, consumption of fluoroquinolones for production animals (pigs, poultry, and cattle) was reduced from 114 kg in 2001 to 18 kg in 2005 (*12*).

### New Guidelines for Veterinary Prescription of Antimicrobial Agents for Pigs

Pigs constitute the largest volume of animals produced in Denmark. In 2005, a total of 25.7 millions pigs were produced, and pig production accounted for 82% of the total veterinary consumption of antimicrobial agents (*12*). From 2002 through 2004, antimicrobial use increased by 27%; large regional differences indicated that the increase was related to local factors (*27*). To reduce the use of antimicrobial agents and encourage specific substitutions of different agents in pig production, in 2005 the Danish Veterinary and Food Administration published a set of new guidelines for veterinary prescription of antimicrobial agents for pigs. The guidelines were developed in cooperation with relevant national institutions. They exclude quinolones and cephalosporins from the list of recommended agents. The effect of these guidelines on antimicrobial agent use in pig production cannot yet be determined.

### Increased Resistance in Zoonotic Bacteria from Imported Meat

Since 1998, levels of resistance have been higher in *Salmonella* isolates from imported food than in isolates from Danish food (*28*). In 2005, the occurrence of resistance in *Salmonella* Typhimurium isolated from imported pork generally exceeded that of corresponding isolates from Denmark (*12*). A similar tendency was observed for *Campylobacter jejuni* isolated from poultry meat: imported poultry meat showed a higher resistance frequency than Danish poultry meat. The high frequency of antimicrobial agent resistance in *Salmonella* from imported pork and in *Campylobacter* spp. from imported poultry meat probably reflects differences in the use of veterinary antimicrobial agents in the countries of origin as compared with Denmark (*12*). The higher occurrence of antimicrobial agent resistance in zoonotic bacteria in imported meat products is important because a large part of meat consumed in Denmark is of foreign origin. In November 2006, case-by-case evaluation of the safety of imported meat was implemented.

In the DANMAP 2000 report, higher levels of resistance in travel-associated *Salmonella* and *Campylobacter* infections were reported for the first time (*29*). Increased ciprofloxacin resistance in isolates from infections acquired abroad was particularly significant. Similarly, *C. jejuni* isolates from human infections acquired abroad generally had a higher frequency of resistance to ciprofloxacin and tetracycline (*12*). From 2001 through 2005, the number of long holidays (>4 overnight stays) for Danish travelers increased by 14%. The most popular destinations were Spain, France, and Italy, which accounted for >30% of the long holidays abroad in 2005. Travel to the southern part of Europe might be associated with risk of acquiring infections due to resistant zoonotic pathogens because *Salmonella* and *Campylobacter* spp. isolated from meat produced in southern Europe are generally more resistant to antimicrobial agents than isolates obtained from meat produced in Denmark (*30*). Thereby, foodborne zoonotic infections acquired in southern Europe may offer limited therapeutic options. Another high-risk area for acquiring antimicrobial-resistant foodborne zoonotic infections is Asia (*31*).

### Increased Human Consumption of Therapeutic Antimicrobial Agents

Since the early 1980s, data on use of antimicrobial agents for human therapy have been available from pharmaceutical industry sources or from the Danish Medicines Agency. DANMAP systematically reports these data in conjunction with resistance data, which has enabled identification of specific problems linked to antimicrobial consumption for human therapy (Table).

Increasing use of antimicrobial agents by humans, both outside and inside hospitals, was reported by DANMAP in 2000 (*29*). At the time, antimicrobial consumption by outpatients was among the lowest in Europe and was similar to that in Germany, Sweden, and Austria (*32*). Since then the increase has continued for outpatients (data not shown) and hospitalized patients. The mean antimicrobial consumption in hospitals has increased by 39%, from 421 defined daily doses (DDD)/1,000 bed-days in 1997 to 585 DDD/1,000 bed-days in 2004 (*14*). Much of this increase can be explained by increased hospital activity, i.e., an increased number of patients treated in hospitals concomitant with shorter lengths of hospital stay. Another reason for the increased consumption has been an increase in doses, based on better understanding of the pharmacokinetic and pharmacodynamic properties of antimicrobial agents. Still, parts of the increase are unaccounted for, and much remains to be understood to explain this increase in antimicrobial consumption in humans and how it may be controlled. In addition to increased total use of antimicrobial agents, newer antimicrobial agents are being used, e.g., cephalosporins, fluoroquinolones, and carbapenems, at the expense of extended-spectrum penicillins (except pivmecillinam), aminoglycosides, and macrolides (*14*). Although use of quinolones remains lower in Denmark than in European countries (*33*), a small but statistically significant increase in the frequency of ciprofloxacin-resistant *E. coli* isolates from urine has been observed since 2003 in primary healthcare facilities and in hospitals. The increase in ciprofloxacin resistance has occurred concurrently with a recent increase in the consumption of fluoroquinolones, primarily ciprofloxacin (*14**,**34*).

### Publication of MRSA Guidelines

An increase in MRSA cases was observed in 2000 (97 cases) and continued in 2001 (104 cases) (*29**,**35*). The number of MRSA cases, including infection and colonization, reached 856 in 2005 (*12*). As a response to this increase, new national guidelines for the control and prevention of MRSA were issued by the National Board of Health (*35*). The guidelines enforce use of the search-and-destroy policy in hospitals as well as in other healthcare institutions such as nursing homes. Additionally, to maintain a low colonization pressure in Denmark and thus reduce cross-transmission, all MRSA-positive persons are offered eradication treatment (*35*). The guidelines also recommend that MRSA-positive persons be given a personal “MRSA card,” which must be shown at each contact with healthcare providers to ensure proper treatment and to prevent further transmission (*35*). To better monitor the new MRSA situation in Denmark and to facilitate implementation of control measures in connection with outbreaks, reporting MRSA cases has been mandatory since November 1, 2006 (*16*).

### Increased Pneumococcal Resistance to Macrolides

In 2000, susceptibility testing performed on pneumococcal isolates from blood and cerebrospinal fluid sent to SSI showed that the frequency of erythromycin resistance in pneumococci slowly increased from ≈0% in 1990 to 3.4% in 1999 (*36*). This increase in macrolide resistance of pneumococci was probably related to a relative high consumption of macrolides combined with a change in the distribution of the macrolides used (*13*). Since 2000, macrolide resistance in pneumococci from blood and spinal fluid has been ≈5% (*12*).

The Danish experience shows that even if antimicrobial agent consumption is generally low and the frequency of resistance is correspondingly small, a temporary rise in consumption of even a single class of antimicrobial agent can shift this balance in an unfavorable direction. In Denmark, rational antimicrobial therapy is the tool to ensure optimum treatment of patients with bacterial infections and low levels of antimicrobial agent resistance. However, continuous training and efforts are essential to keep general practitioners as well as hospital specialists updated on the rational use of antimicrobial agents (*14*).

## Possible New Areas for DANMAP Monitoring

Unlike the monitoring programs in Sweden and Norway, DANMAP never included bacteria obtained from companion animals (*5*,*6*), and rational therapy guidelines for companion animals have not been promoted or implemented in veterinary university clinics or private veterinary practices. Fluoroquinolone and cephalosporin consumption by companion animals in Denmark is substantial compared with that of food animals (*37*). Considering the shared environment of humans and companion animals, transfer of resistant bacteria or of mobile resistance determinants from companion animals to humans seems possible. Thus, emergence of resistance to fluoroquinolones and cephalosporins in companion animals should be a matter of concern and could be considered a new area for surveillance. Recently, MRSA has been detected in companion animals and in food-producing animals in other countries, the potential importance of which should also be monitored (*38*).

A substantial proportion of human *Salmonella* isolates belongs to serotypes other than *S.* Enteritidis and *S.* Typhimurium. The occurrence of more uncommon serotypes is increasing and in 2005 represented ≈30% of all human *Salmonella* isolates (*12*). Prevalence of antimicrobial agent resistance varies greatly among less frequent *Salmonella* serotypes isolated in Denmark, and specific serotypes showed a high level of resistance (*39*). These *Salmonella* serotypes, as well as other emerging or reemerging pathogens such as *Mycobacterium tuberculosis* (*40*), represent a potential focus area.

In future DANMAP reports, more attention should be given to the presence of extended-spectrum β-lactamase (ESBL)–producing *E. coli* and *Salmonella* from animals and humans. The emergence of ESBL resistance is a new threat for human therapy.

## Conclusions

DANMAP has led to changes in the use of antimicrobial agents in Denmark and other countries. Until now, the effect in Denmark had been seen mostly in animals, but awareness has been raised for humans as well (Table). One of the strengths of DANMAP is cooperation between veterinary and human healthcare providers, thus offering a broad range of viewpoints and professionals. This integrated program was made possible because access to all relevant data and samples that were already systematically collected from animals, food, and humans has been shared. To complete these data, a random sampling of indicator bacteria “from farm to fork” was implemented, which has made follow-up of antimicrobial agent resistance for zoonotic and indicator bacteria possible.

The relationship between antimicrobial agent resistance in the food supply and human foodborne infections is complex. It depends on the level of resistance of bacteria in domestic food, level of resistance in imported food, and influence of travel abroad. The need for surveillance of antimicrobial consumption and resistance in animals and humans is universal because food, humans, and even livestock travel. Solid scientific data are needed for an evidence-based debate and to facilitate further regulation regarding antimicrobial agent resistance and consumption.
